# Stachydrine Ameliorates Uterine Hypercontractility in Primary Dysmenorrhea by Targeting the COX-2/PGF2α Pathway

**DOI:** 10.3390/cimb47110961

**Published:** 2025-11-19

**Authors:** Yongfeng Cheng, Shuo Chen, Dianjie Cao, Hairu Cheng, Siyuan Chen, Yi Shu, Yue Wang, Zhiwu Chen

**Affiliations:** 1 Clinical College of Anhui Medical University, Hefei 230012, China; yfcheng@ustc.edu.cn (Y.C.); caodianjie@aycc.edu.cn (D.C.); 13093559207@163.com (S.C.); 17764434861@163.com (Y.S.); 2 School of Traditional Chinese Medicine, Anhui University of Chinese Medicine, Hefei 230038, China; chasemoon@aliyun.com; 3 Department of Pharmacology, School of Basic Medical Sciences, Anhui Medical University, Hefei 230032, China; 4 School of Life Science, University of Science and Technology of China, Hefei 230027, China; agswy@mail.ustc.edu.cn

**Keywords:** stachydrine, PDM, COX-2, PGF2α, Indo, uterine contractions

## Abstract

Primary dysmenorrhea (PDM) is a typical gynecologic disease in which uterine contractions and inflammation cause pain. Stachydrine (Sta) possesses multiple pharmacological activities but its effect on PDM has not yet been clarified. In vitro uterine contraction and oxytocin (OT)-induced PDM mouse models were used to evaluate the effect of Sta. Sta (10^−6.5^ to 10^−4^ mol/L) dose-dependently inhibited spontaneous and OT-induced uterine contractions, with maximum inhibition rates of 47.1% and 40.4%, respectively. This effect was reversed by N-nitro-L-arginine (L-NAME) and indomethacin (Indo), suggesting the involvement of the nitric oxide and prostaglandin pathways. In vivo, Sta (20, 10, 5 mg/kg) significantly reduced writhing episodes, prolonged latency to the first response, and alleviated OT-induced uterine damage and inflammation. Additionally, Sta downregulated cyclooxygenase-2 (COX-2) expression in uterine tissue and decreased serum malondialdehyde (MDA) and prostaglandin F_2_α (PGF2α) levels. These findings suggest that Sta alleviates PDM by modulating the COX-2/PGF2α pathway, inhibiting uterine contractions, and reducing inflammation and oxidative stress, making it a promising therapeutic candidate for PDM.

## 1. Introduction

Primary dysmenorrhea (PDM) usually arises in young women with its pain symptoms typically occurring in the lower abdomen at the onset of menses. The principal mechanisms involved in the pathophysiology of PDM is excessive contraction of the uterine smooth muscle and elevated levels of prostaglandins (PGs) in the endometrium causing ischemia and pain [1,2]. Furthermore, oxidative stress and inflammation have important roles in initiating and sustaining the pathological process of PDM [3,4,5].

Conventional treatments, including oral contraceptives and nonsteroidal anti-inflammatory drugs (NSAIDs) [6,7], are often associated with adverse effects such as gastrointestinal irritation and cardiovascular complications [8]. This underscores the need for alternative therapeutic strategies, particularly those involving natural compounds with multi-targeted actions and minimal adverse effects.

***Leonurus japonicus Houtt***, commonly known as motherwort, is a traditional herbal medicine extensively used for gynecological conditions such as menstrual irregularities and dysmenorrhea [9,10]. As reviewed by Shang et al., its complex chemical composition underpins a range of pharmacological activities, including significant uterine excitatory, cardiovascular protective, anti-inflammatory, and antioxidant effects. Among its components [11], stachydrine (Sta) is one of key active component that exhibits anti-inflammatory and antioxidant effects [12,13,14,15]. However, the specific mechanisms through which Sta exerts therapeutic effects in PDM remain inadequately explored.

In this study, we assessed Sta’s effects on both spontaneous and OT-induced uterine contractions in vitro. Furthermore, an in vivo OT-induced PDM mouse model was utilized to investigate the therapeutic effects of Sta by measuring uterine contractions [1,2,16,17,18] oxidative stress markers, and inflammatory mediators, and analyzing the histopathological changes [19,20]. These results could reveal Sta’s potential as a therapeutic for PDM.

## 2. Materials and Methods

### 2.1. Materials and Animals

#### 2.1.1. Chemicals and Reagents

Stachydrine (Sta) was sourced from Anhui Traditional Chinese Medical College (Hefei, China). Oxytocin (1302031) was obtained from Henan Furen Pharmaceutical Co., Ltd. (Zhengzhou, China). Arginine analog L-NAME (N5501), a nitric oxide synthase (NOS) inhibitor, and indomethacin (Indo, 53861) were supplied by Sigma Co., Ltd. (St. Louis, MO, USA). Estradiol benzoate (C20230927) was obtained from the Ningbo Second Hormone Factory (Ningbo, China). Malondialdehyde (MDA, A003-1-2) and superoxide dismutase (SOD. A001-3-2) assay kits were procured from Nanjing Jiancheng Biotechnology Institute (Nanjing, China). PGF2α ELISA kits (JL13273) were provided by Jianglai Biotechnology Co., Ltd. (Shanghai, China), while mouse COX-2 ELISA kits (ml037732) were sourced from Shanghai Enzyme-Linked Biotechnology Co., Ltd. (Shanghai, China). All chemical reagents were analytical grade and were dissolved in military injection water. Stock solutions of the drugs were prepared using distilled water at the required concentrations and stored at 4 °C.

#### 2.1.2. Animal Treatment

Female BALB/c mice (body weight 18–22 g) were purchased from the Experimental Animal Center of Anhui Medical University (Hefei, China). All animal experiments were approved by the Laboratory Animal Welfare and Ethics Committee of the Clinical College of Anhui Medical University (Approval number: LCYXY0005; Date: 2 Apr 2024). Mice were randomly assigned into six groups (*n* = 6/group): vehicle, model, Sta-L (5 mg/kg), Sta-M (10 mg/kg), Sta-H (20 mg/kg), and ibuprofen (100 mg/kg). The vehicle group mice were subcutaneously injected with saline, and the other five groups were subcutaneously injected with 10 mg/kg estradiol benzoate once per day for 7 d. On the 4th day, after the estradiol benzoate treatment, the Sta-L, Sta-M, and Sta-H groups were administered Sta at their respective dose through intragastric instillation while the model and positive control groups were injected with an equivalent volume of saline and ibuprofen, respectively. On day 7, all the mice were given an intraperitoneal dose of oxytocin (OT; 100 U/kg) to induce writhing in the mice (except for those in the vehicle group). The mice were housed in standard cages with bedding in a 12 h light cycle at a temperature of 22 ± 2 °C and humidity of 50 ± 10%. All the animals had free access to food and water.

### 2.2. Uterine Strip Preparation and Incubation

Female mice were sacrificed by decapitation, and their uteri were carefully excised and cut into two strips, each approximately 2–3 cm in length. The excised uterine tissue was immediately placed in a plate containing Locke–Ringer solution [21,22], and incubated for 30 min at 4 °C. The Locke–Ringer solution had the following composition: 9.0 g/L NaCl, 0.42 g/L KCl, 0.12 g/L CaCl_2_, 0.2 g/L NaHCO_3_, and 2.0 g/L glucose. Next, the uterine strips were immersed in separate organ baths at 34 ± 0.5 °C and bubbled with a gas mixture (95% O_2_ + 5% CO_2_). Each uterine strip was subjected to an initial tonus of 1 g and was allowed to equilibrate for approximately 30 min. Contractile responses were recorded using a bioinformatics collection system (BL-420E, Chengdu Taimeng Software Co., Ltd., Chengdu, China). After the equilibration period, the drugs were added to Krebs solution in 10-min intervals. The frequency and tension of uterine contractions were measured throughout the experiment.

### 2.3. Spontaneous Uterine Contraction Assays

Isolated uterine strips were placed in Locke’s (34 ± 0.5 °C) solution for 30 min. Sta was added in a cumulative manner every 10 min, with the final concentrations ranging from 10^−6.5^ to 10^−4^ mol/L. The control group was maintained without treatment for 60 min. The frequency and tension of spontaneous uterine contractions were recorded every 10 min throughout the experiment. The frequency relaxation percentage was calculated for spontaneous contractions using the following formula:

$$\mathrm{Relaxation}(\%)\;=\;\frac{A-B}{A}\times 100\%$$

where *A* is the frequency of spontaneous uterine contractions in 10 min before the drug treatment, and *B* is the frequency of uterine contractions after treatment with Sta for 10 min (10^−6.5^ to 10^−4^ mol/L). The percentage of tension relaxation was also calculated.

### 2.4. OT-Induced Uterine Contraction Assays

Uterine strips were pre-equilibrated in Locke–Ringer solution for 30 min at 34 ± 0.5 °C. Following equilibration, the strips were pre-treated with oxytocin (OT) at a concentration of 20 U/L for 10 min. Sta then was added cumulatively every 10 min to a maximum final concentration of 10^−6.5^ to 10^−4^ mol/L. For the first 60 min, the OT group (control) was not treated with Sta. The frequency and tension of the OT-induced uterine contractions were measured at each 10 min interval. Relaxation was calculated using the same equation used for spontaneous contractions.

### 2.5. Effect of Pretreatment with Indo or L-NAME on OT-Induced Uterine Contractions

Uterine strips were pre-incubated in Locke–Ringer solution with either L-NAME (1 × 10^−4^ mol/L) or Indo (6 × 10^−5^ mol/L) for 30 min, followed by treatment with OT (20 U/L) for 10 min. Subsequently, Sta was added in a cumulative manner every 10 min, with the final concentrations ranging from 10^−6.5^ to 10^−4^ mol/L. The OT + Indo and OT + L-NAME groups were maintained for 60 min. The uterine contraction frequency was recorded every 10 min throughout the experiment.

### 2.6. OT-Induced Writhing Assessment

A uterine OT-induced contraction model [23,24] was developed as follows. Forty-eight female BALB/c mice were allocated into 6 groups: a vehicle control, an OT model, three Sta groups (5, 10, and 20 mg/kg), and an ibuprofen positive control (100 mg/kg). All groups, except the vehicle control, underwent subcutaneous estradiol benzoate administration for 7 consecutive days. On the fourth day, 30 min after the estradiol benzoate treatment, the mice in the Sta and ibuprofen groups were orally administered Sta (5, 10, or 20 mg/kg) or ibuprofen (100 mg/kg), respectively. One hour later, OT (100 U/kg) was intraperitoneally injected. The duration of the writhing responses and the latency to body twisting were documented for 30 min post-OT administration. Plasma samples for MDA and SOD measurements were collected using the retro-orbital blood sampling method. Subsequently, the animals were euthanized, and uterine tissues were harvested for further evaluation.

### 2.7. Histopathological and Immunohistochemical Analyses

Uterine strips were immersed in formalin (10% buffer) for 24 h, and dehydrated in a graded ethanol series. The uterine strips were then embedded in paraffin, cut into sections about 5 μm thick, mounted on slides, deparaffinized, and stained with hematoxylin and eosin (H&E) for routine histopathological evaluation using an Olympus DX45 Microscope (Tokyo, Japan) with the researcher blinded to the treatment details.

### 2.8. Biochemical Analysis of Serum and Uterine Tissue

The serum MDA and SOD levels were quantified according to the experimental guidelines. The levels of COX-2 and PGF2α in uterine tissue were measured using enzyme-linked immunosorbent assay kits.

### 2.9. Statistical Analysis

GraphPad Prism software 8.0 was used to analyze the data. All data are presented as the mean ± standard deviation (SD). One-way ANOVA and multiple *t* tests were applied to analyze data, with *p*-values less than 0.05, 0.01, and 0.001 indicating significant, highly significant, and exceedingly significant differences, respectively.

## 3. Results

### 3.1. Sta Reduced the Spontaneous Contraction Frequency in Isolated Uteri

**Figure 1:**
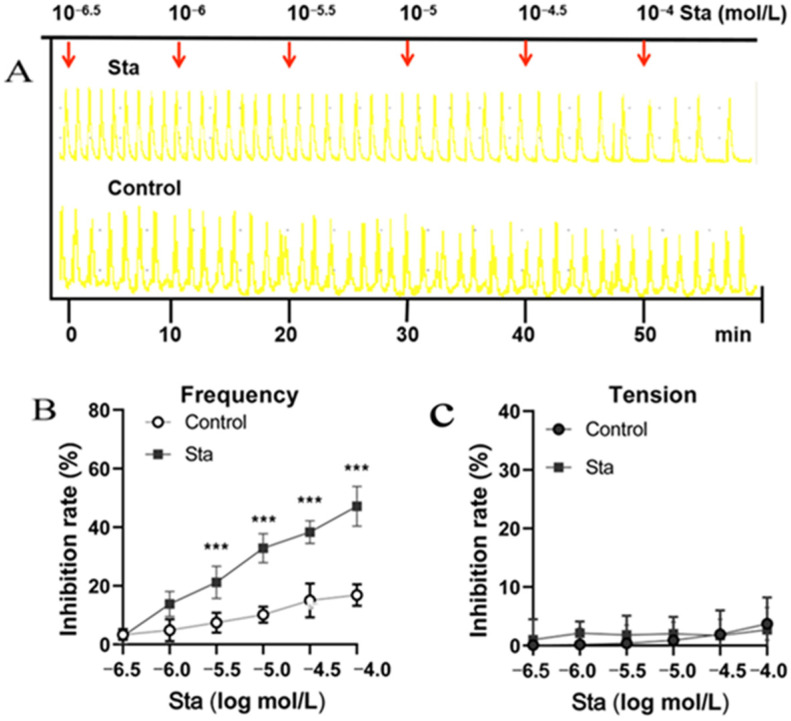
The impact of stachydrine (Sta) on spontaneous uterine contractions in vitro. The uterine strips were incubated in Locke–Ringer solution at 4 °C and then were transferred to isolated organ baths maintained at 34 ± 0.5 °C and bubbled with a gas mixture of 95% O_2_ and 5% CO_2_. Sta was added in a cumulative manner (10^−6.5^ to 10^−4^ mol/L) every 10 min. (**A**) Representative traces illustrating the relaxant effects of Sta on spontaneous contractions in isolated mouse uteri. The frequency (**B**) and intensity (**C**) of the contractions were measured every 10 min. Error bars indicate standard deviation (SD); *n* = 6. A statistically significant difference of *** *p* < 0.001 was observed between the Sta and control group.

The spontaneous contraction trajectories of isolated uteri and the timing of drug administration are presented in Figure 1A. The initial concentration of Sta was 10^−6.5.^mol/L; Sta was added every 10 min to form a concentration series of 10^−6.5^, 10^−6^, 10^−5.5^, 10^−5^, 10^−4.5^, and 10^−4^ mol/L. Compared with the spontaneous contractions of the control uteri, Sta inhibited the contractions. Relaxation was determined as the percentage decrease in spontaneous contractions. As shown in Figure 1B, Sta at concentrations of 10^−6.5^, 10^−6^, 10^−5.5^, 10^−5^, 10^−4.5^, and 10^−4^ mol/L reduced the contraction frequency by 13.8%, 21.2%, 32.8%, 38.3%, and 47.1%, respectively, in a dose-dependent manner. However, Sta had no significant effect on the tension of spontaneous contractions (Figure 1C).

### 3.2. Sta Reduced the Oxytocin (OT)-Induced Contraction Frequency in Isolated Uteri

**Figure 2:**
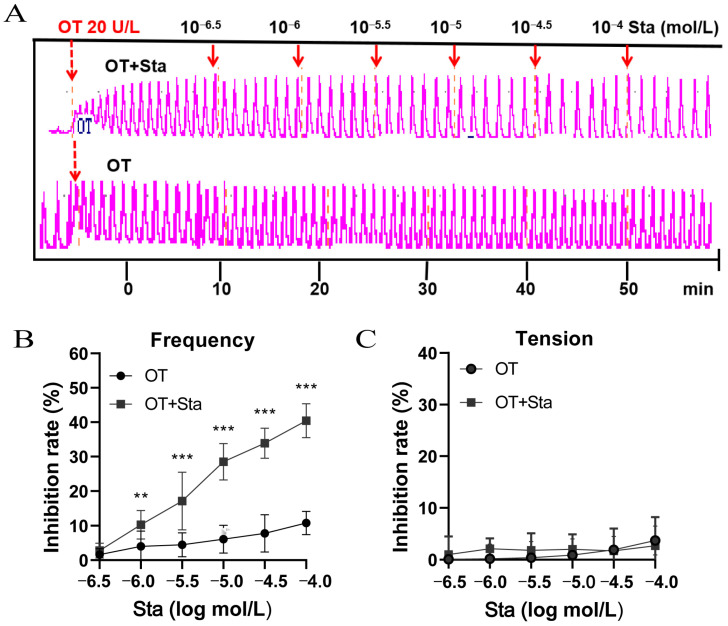
The impact of Sta on oxytocin (OT)-induced uterine contractions in vitro. The uterine strips were transferred to isolated organ baths and treated with OT (20 U/L) for 10 min. Sta was cumulatively administered at concentrations ranging from 10^−6.5^ to 10^−4^ mol/L every 10 min. (**A**) Representative traces illustrating relaxant action of Sta on isolated mouse uteri. The frequency (**B**) and intensity (**C**) of the uterine contractions were recorded at each 10 min interval. Error bars represent the SD; *n* = 6. ** *p*< 0.01 and *** *p* < 0.001 vs. the OT group.

OT induced intense uterine contractions, significantly enhancing contractility at a dose of 20 U/L. The effects of Sta on OT-induced uterine contractions and administration timing are shown in Figure 2A. As illustrated in Figure 2B, Sta at concentrations of 10^−6.5^, 10^−6^, 10^−5.5^, 10^−5^, 10^−4.5^, and 10^−4^ mol/L reduced the contraction frequency by 10.2%, 17.1%, 28.5%, 33.9%, and 40.4%, respectively, in a dose-dependent manner. Sta did not alter the contraction tension (Figure 2C).

### 3.3. Both L-NAME and Indo Attenuated the Inhibition of OT-Induced Uterine Contractions by Sta

**Figure 3:**
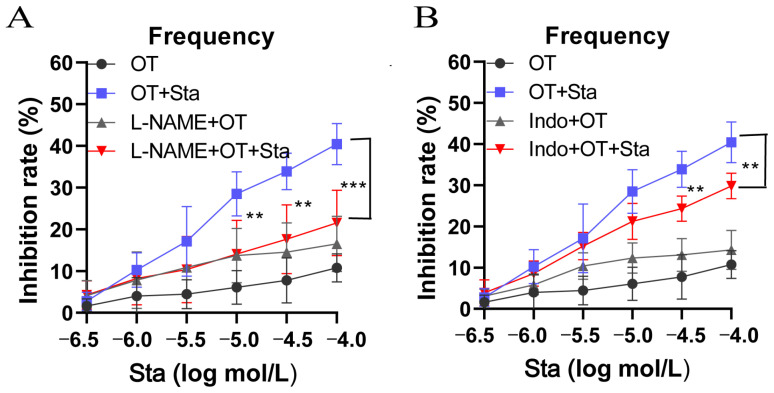
N-nitro-L-arginine (L-NAME) and indomethacin (Indo) attenuated the Sta-induced reduction in OT-mediated contraction frequency. Uterine strips were pre-incubated for approximately 30 min with L-NAME (a nitric oxide synthase inhibitor) or Indo (a cyclooxygenase inhibitor), followed by OT (20 U/L) treatment for 10 min. Sta was then applied cumulatively (10^−6.5^ to 10^−4^ mol/L) at 10 min intervals. The contraction frequency in the L-NAME (**A**) and the Indo (**B**) groups. Error bars represent the SD; *n* = 6. ** *p*< 0.01 and *** *p* < 0.001 vs. the OT + Sta group.

The effects of L-NAME, a nitric oxide synthase inhibitor, and Indo, a cyclooxygenase inhibitor, on Sta inhibition of uterine contractions were assessed in this study. As shown in Figure 3A, compared with the OT + Sta groups, the inhibitory rates of the L-NAME + OT + Sta groups (10^−5^, 10^−4.5^, and 10^−4^ mol/L) were markedly reduced. L-NAME significantly diminished Sta’s inhibitory effect on the frequency of OT-induced uterine contractions (*p* < 0.01). Similarly, Indo also attenuated the inhibitory effect of Sta (Figure 3B). The Indo + OT + Sta groups (10^−4.5^ and 10^−4^ mol/L) had significantly reduced contraction frequencies (*p* < 0.01).

### 3.4. Effect of Sta on OT-Induced Writhing

**Figure 4:**
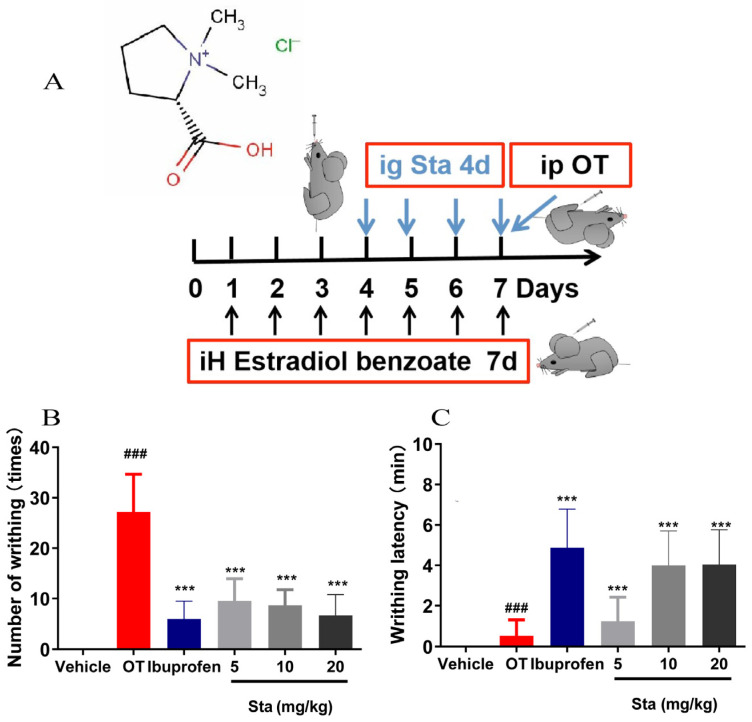
Sta reduced the OT-induced writhing behavior in a mouse model pre-treated with estradiol benzoate. (**A**) Schematic representation of the experimental design. Mice were subcutaneously injected with estradiol benzoate (2.5 mg/kg/day) for 7 consecutive days. From day 4 to day 7, the mice were orally administered Sta at varying doses (5, 10, and 20 mg/kg/day). On day 7, an intraperitoneal injection of OT (100 U/kg) was given to induce writhing behavior. The number of writhing events (**B**) and writhing latency (**C**) were immediately recorded over a 30-min period. Data are expressed as the mean ± SD (*n* = 6). ### *p* < 0.001 vs. the vehicle group, *** *p* < 0.001 vs. the OT group.

The OT-induced writhing test, a classic tool for evaluating analgesic effects in primary dysmenorrhea (PDM), was used. The drug administration protocol is shown in Figure 4A. In the OT group, the number of writhing episodes was 27.2 ± 7.5 times within 30 min. In contrast, Sta at doses of 5, 10, and 20 mg/kg significantly reduced the number of writhing episodes to 9.5 ± 4.4, 8.7 ± 3.1, and 6.7 ± 4.1, respectively (*p* < 0.001, Figure 4B). Additionally, the latency periods of the Sta groups (5, 10, and 20 mg/kg) were 1.2 ± 1.1, 4.0 ± 1.7, and 4.0 ± 1.7 min, significantly longer than that of the OT group (0.5 ± 0.8 min, *p* < 0.001) (Figure 4C).

### 3.5. Impact of Sta on Uterus Gross Morphology and Histopathology

**Figure 5:**
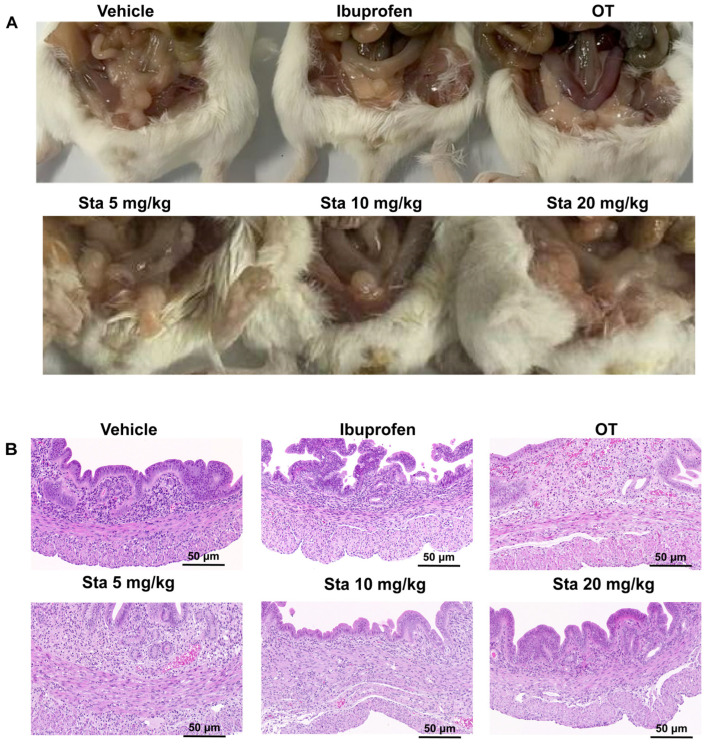
Histological examination of mouse uterine tissues from the vehicle, OT, ibuprofen (100 mg/kg), and Sta treatment (5, 10, and 20 mg/kg) groups. (**A**) Representative images of uterine tissues. (**B**) H&E staining of mouse uterine tissues (magnification: 50×) highlighting histological alterations, such as inflammatory cell infiltration, endometrial congestion, and edema.

As depicted in Figure 5A, the OT-treated group, uterine congestion and edema could be easily observed. Ibuprofen and Sta at all tested doses (5, 10, and 20 mg/kg) relieved these inflammation-related symptoms, with Sta treatment showing dose-dependent effects. The histological investigation of uterine tissues stained with H & E. (Figure 5B), noticeable showed infiltration of inflammatory cells, a damaged endometrial layer, and cellular edema in the OT-treated group. The inflammatory cell infiltration and damaged endometrial layer both showed significant improvement after treatment with ibuprofen or Sta. Sta at high doses (10 and 20 mg/kg) had effects similar to those of ibuprofen.

### 3.6. Effect of Sta on COX-2 Expression in Uterine Tissues

**Figure 6:**
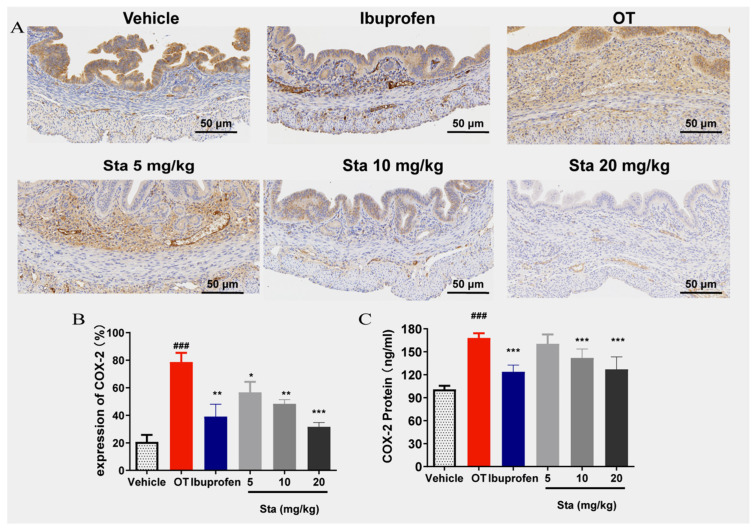
(**A**) Representative immunohistochemical sections showing cyclooxygenase-2 (COX-2) expression in the vehicle, OT, ibuprofen (100 mg/kg), and Sta treatment (5, 10, and 20 mg/kg) groups (magnification: 50×). (**B**) Quantification of COX-2 expression levels in uterine tissues using immunohistochemistry. (**C**) COX-2 protein levels in uterine tissue measured using ELISAs. Data are expressed as the mean ± SD *(n* = 6). ### *p* < 0.001 vs. the vehicle group; * *p* < 0.05, ** *p* < 0.01, *** *p* < 0.001.

As shown in Figure 6A,B, COX-2 expression was significantly elevated in the OT group compared with the vehicle group (*p* < 0.05). However, Sta at doses of 5, 10, and 20 mg/kg significantly reduced COX-2 expression in a dose-dependent manner. The quantification of COX-2 protein concentrations within uterine tissue using enzyme-linked immunosorbent assays (ELISAs) confirmed these findings (Figure 6C).

### 3.7. Effects of Sta on MDA, SOD, and PGF2α Levels

**Figure 7:**
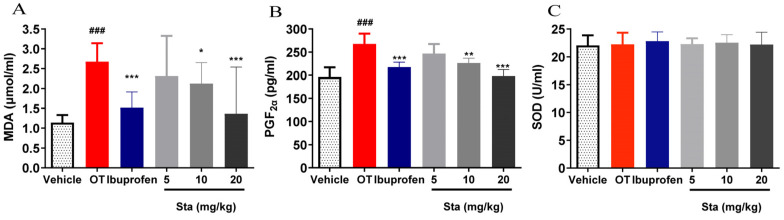
Effects of Sta on malondialdehyde (MDA) levels (**A**) and superoxide dismutase (SOD) activity (**C**) in the serum, and prostaglandin F2α (PGF2α) levels (**B**) in the uterine tissue of OT-induced mice. Vertical bars represent the standard deviation (SD). ### *p* < 0.001 vs. the vehicle group; * *p* < 0.05, ** *p* < 0.01, and *** *p* < 0.001, vs. the OT group.

OT administration significantly increased MDA levels in the serum and PGF2α levels in the uterus. Treatment with Sta at doses of 10 and 20 mg/kg effectively reduced both MDA and PGF2α levels (*p* < 0.05; Figure 7A,B). However, there were no significant differences in serum SOD levels between the groups, including those receiving Sta treatment (Figure 7C).

## 4. Discussion

Oxytocin (OT) induces strong and sustained contractions analogous to those in dysmenorrhea, and is are widely employed in in vitro models used to evaluate the efficacy of drugs for this condition [20]. Here, we used this model and demonstrated that stachydrine (Sta) effectively reduces uterine contraction frequency in both spontaneous and OT-induced models without affecting contraction tension. We speculate that Sta may specifically modulate uterine pacemaker activity, thereby prolonging contraction intervals and reducing the frequency without significantly affecting contraction tension [25]. This is likely because it does not alter the peak intracellular calcium release or the calcium sensitivity of the contractile proteins required for generating strong contractile forces [26]. Such target specificity results in the “decoupling” between contraction frequency and tension. Based on our research findings that pretreatment of isolated uteri with Indo and L-NAME can reduce the OT-induced increase in uterine contraction frequency, this study suggests that Sta may inhibit the OT-induced increase in uterine contraction frequency by suppressing the activity of NOS and COX expression.

The OT-induced primary dysmenorrhea (PDM) model in mice is a widely recognized pharmacodynamic experimental model for dysmenorrhea research and is commonly used in studies of primary dysmenorrhea [27]. The main clinical symptom of PMD is severe acute abdominal pain; therefore, the writhing response is used as a primary indicator to evaluate the efficacy of experimental drugs. This study found that compared with the normal group, the number of writhing episodes within 30 min significantly increased and the latency period decreased in the model group. Both the ibuprofen positive control and Sta groups showed a reduced number of writhing responses within 30 min and a prolonged pain latency period, indicating that Sta can antagonize OT-induced acute abdominal pain in mice. The histopathological analysis demonstrated that Sta mitigated OT-induced damage to uterine tissue, reducing inflammatory cell infiltration and structural disorganization.

PDM may be related to transient ischemia in the myometrium and endometrium resulting from paroxysmal contractions of the endometrial smooth muscle that compress intramural blood vessels, leading to an increase in free radicals [28]. MDA, as a key product of lipid peroxidation metabolism, may cause cellular damage, and its level can indirectly reflect the extent of free radical-induced damage to uterine cells. Sta reduced serum malondialdehyde (MDA) levels, indicating its antioxidative potential, while having no effect on serum superoxide dismutase (SOD). The lack of an effect on SOD suggests that its antioxidant mechanism may be independent of this particular enzyme and that it may affect other antioxidant systems like glutathione.

Our study identified arachidonic acid (AA) metabolism and oxytocin signaling as the central pathways in primary dysmenorrhea (PDM) pathogenesis. AA serves as the key precursor for prostaglandin (PG) synthesis, and its metabolic dysregulation contributes significantly to various physiological and pathological processes. The endometrium represents a major site of PG production. Evidence indicates that PDM pathogenesis involves hypercontractility of the uterus driven by excessive endometrial prostaglandin secretion. Prostaglandin biosynthesis has been shown to play a role in pain mechanisms in multiple pain models [29]. Specifically, PGF2α promotes uterine contractions and induces ischemic conditions, directly contributing to menstrual pain.

In our experimental models, uterine tissue PGF2α levels were significantly elevated compared to normal controls. Both ibuprofen and Sta treatments substantially reduced these elevated PGF2α levels. Dysmenorrhea is known to have an inflammatory component, with prostaglandin overproduction representing a fundamental mechanism in primary dysmenorrhea. Cyclooxygenase (COX), particularly the inducible COX-2 isoform, functions as the rate-limiting enzyme in PG biosynthesis [30]. COX-2 overexpression leads to excessive prostaglandin production, subsequently exacerbating uterine contractions and pain perception.

Consistent with this mechanism, COX-2 expression in uterine tissues was markedly higher in the OT-induced model groups compared to the normal controls. Both the ibuprofen and Sta treatments effectively suppressed this elevated COX-2 expression. These collective findings suggest that Sta’s therapeutic efficacy may be mediated through inhibition of the COX-2/PGF2α signaling axis, highlighting its pathological relevance in PDM and its potential as a mechanistic target for therapeutic interventions.

These findings support the existing research showing a role for oxidative stress and inflammation in PDM pathophysiology. Sta’s ability to modulate uterine contractility, along with its anti-inflammatory and antioxidative effects, highlights its potential as a therapeutic for PDM. Further clinical studies are needed to confirm these preclinical results and assess Sta’s safety and efficacy in PDM management.

## 5. Conclusions

In conclusion, this study provides new insights into the therapeutic potential of stachydrine (Sta) for the management of primary dysmenorrhea (PDM). Our results demonstrate that Sta effectively reduces the frequency of uterine contractions, alleviates pain behaviors, and mitigates uterine tissue damage associated with PDM. Notably, Sta’s modulation of oxidative stress and inflammatory pathways, particularly through reductions in COX-2 expression and PGF2α levels, highlights its complex mechanism of action. These findings suggest that Sta, a bioactive compound derived from motherwort, could serve as a promising alternative to conventional treatments for PDM, offering potential benefits with minimal side effects. Further clinical studies are needed to confirm these preclinical findings and to evaluate the safety, efficacy, and optimal dosing of Sta for PDM in women.

## Data Availability

The raw data supporting the conclusions of this article will be made available by the authors on request.

## References

[1] Sun L., Liu L., Li J., Lv Y., Zong S., Zhou J., Wang Z., Kou J., Xiao W. (2017). The essential oil from the twigs of *Cinnamomum cassia* Presl inhibits oxytocin-induced uterine contraction in vitro and in vivo. J. Ethnopharmacol..

[2] Dawood M.Y. (2006). Primary Dysmenorrhea: Advances in Pathogenesis and Management. Obstet. Gynecol..

[3] Eroglu O., Comertpay E., Vural S., Badem N.D., IşBaşaran P., Neşelioğlu S., Erel Ö., Deniz T. (2022). Diagnostic value of oxidative stress markers in patients presenting with primary dysmenorrhea to the emergency department. Niger. J. Clin. Pract..

[4] Kaplan Ö., Nazıroğlu M., Güney M., Aykur M. (2013). Non-steroidal anti-inflammatory drug modulates oxidative stress and calcium ion levels in the neutrophils of patients with primary dysmenorrhea. J. Reprod. Immunol..

[5] Su S., Hua Y., Wang Y., Gu W., Zhou W., Duan J.-A., Jiang H., Chen T., Tang Y. (2012). Evaluation of the anti-inflammatory and analgesic properties of individual and combined extracts from *Commiphora myrrha*, and *Boswellia carterii*. J. Ethnopharmacol..

[6] Itani R., Soubra L., Karout S., Rahme D., Karout L., Khojah H.M.J. (2022). Primary Dysmenorrhea: Pathophysiology, Diagnosis, and Treatment Updates. Korean J. Fam. Med..

[7] Ferries-Rowe E., Corey E., Archer J.S. (2020). Primary Dysmenorrhea: Diagnosis and Therapy. Obstet. Gynecol..

[8] Abdellatif K.R., Abdelall E.K., Bakr R.B. (2017). Nitric Oxide-NASIDS Donor Prodrugs as Hybrid Safe Anti-inflammatory Agents. Curr. Top. Med. Chem..

[9] Miao L., Zhou Q., Peng C., Liu Z., Xiong L. (2019). *Leonurus japonicus* (Chinese motherwort), an excellent traditional medicine for obstetrical and gynecological diseases: A comprehensive overview. Biomed. Pharmacother..

[10] Cheng F., Zhou Y., Wang M., Guo C., Cao Z., Liu W., Zhang J., Wang Y., Wang Y., Zhang L. (2020). A review of pharmacological and pharmacokinetic properties of stachydrine. Pharmacol. Res..

[11] Shang X., Pan H., Wang X., He H., Li M. (2014). *Leonurus japonicus* Houtt.: Ethnopharmacology, phytochemistry and pharmacology of an important traditional Chinese medicine. J. Ethnopharmacol..

[12] Ahmed S.A., Manna P., Borah J.C. (2024). Stachydrine, a pyrrole alkaloid with promising therapeutic potential against metabolic syndrome and associated organ dysfunction. RSC Med. Chem..

[13] Liao L., Tang Y., Li B., Tang J., Xu H., Zhao K., Zhang X. (2023). Stachydrine, a potential drug for the treatment of cardiovascular system and central nervous system diseases. Biomed. Pharmacother..

[14] He Z., Li P., Liu P., Xu P. (2024). Exploring stachydrine: From natural occurrence to biological activities and metabolic pathways. Front. Plant Sci..

[15] Liu F., Yu H., Lee H., Chen C., Liao C. (2024). The Modulation of Phospho-Extracellular Signal-Regulated Kinase and Phospho-Protein Kinase B Signaling Pathways plus Activity of Macrophage-Stimulating Protein Contribute to the Protective Effect of Stachydrine on Acetaminophen-Induced Liver Injury. Int. J. Mol. Sci..

[16] Zhang Q., Zhen J., Hui Z., Meng X., Guan J., Zhang H., Zhang J. (2022). Effect of dexmedetomidine on oxytocin-induced uterine contraction during optimal caesarean section anaesthesia. Basic Clin. Pharmacol. Toxicol..

[17] Kawamata M., Tonomura Y., Kimura T., Sugimoto Y., Yanagisawa T., Nishimori K. (2007). Oxytocin-induced phasic and tonic contractions are modulated by the contractile machinery rather than the quantity of oxytocin receptor. Am. J. Physiol. Endocrinol. Metab..

[18] Cretoiu S.M., Simionescu A.A., Caravia L., Curici A., Cretoiu D., Popescu L.M. (2011). Complex effects of imatinib on spontaneous and oxytocin-induced contractions in human non-pregnant myometrium. Acta Physiol. Hung..

[19] Tang X., Zhou L., Li F., Chen Z. (2024). Regulation of uterine smooth muscle contractions: Implications for dysmenorrhea treatment. Exp. Ther. Med..

[20] Yang X., Tian Y., Liu J., Kou Y., Xie Y., Wang S., Zhao Y. (2023). Peony pollen protects against primary dysmenorrhea in mice by inhibiting inflammatory response and regulating the COX2/PGE2 pathway. Int. J. Mol. Sci..

[21] Campbell P.S., Albright C.W., Wilson J.H., Bridges R.R. (1989). Inhibition of the nuclear localization of [3H]estradiol in rat uterine tissue in vitro. J. Steroid Biochem..

[22] Ichida S. (1986). L-methionine enhances the contractile responses of rat uterine smooth muscle to acetylcholine and high KCl. Jpn. J. Pharmacol..

[23] Wong J., Chiang Y., Shih Y., Chiu C.-H., Chen H.-Y., Shieh T.-M., Wang K.-L., Huang T.-C., Hong Y.-H., Hsia S.-M. (2020). *Salvia sclarea* L. Essential Oil Extract and Its Antioxidative Phytochemical Sclareol Inhibit Oxytocin-Induced Uterine Hypercontraction Dysmenorrhea Model by Inhibiting the Ca^2+^-MLCK-MLC20 Signaling Cascade: An Ex Vivo and In Vivo Study. Antioxidants.

[24] Sun L., Liu L., Zong S., Wang Z., Zhou J., Xu Z., Ding G., Xiao W., Kou J. (2016). Traditional Chinese medicine Guizhi Fuling capsule used for therapy of dysmenorrhea via attenuating uterus contraction. J. Ethnopharmacol..

[25] Sanborn B. (1995). Ion channels and the control of myometrial electrical activity. Semin. Perinatol..

[26] Ni M., Li Y., Wei J., Song Z., Wang H., Yao J., Chen Y.-X., Belke D., Estillore J.P., Wang R. (2023). Increased Ca^2+^ Transient Underlies RyR2-Related Left Ventricular Noncompaction. Circ. Res..

[27] Chen J., Tong R., Sun X. (2013). Establishment of a Mouse Model of Primary Dysmenorrhea Using Progynova Combined with Oxytocin. Chin. J. Exp. Anim..

[28] Chung D., Caruso R. (2006). Potential role for oxidative stress in 2,2’-dichlorobiphenyl-induced inhibition of uterine contractions but notmyometrial gap junctions. Toxicol. Sci..

[29] Bresson E., Boucher-Kovalik S., Chapdelaine P., Madore E., Harvey N., Laberge P.Y., Leboeuf M., Fortier M.A. (2011). The human aldose reductaseAKR1B1 qualifies as the primary prostaglandin F synthase in the endometrium. J. Clin. Endocrinol. Metab..

[30] Zheng W., Li M., Wang Y., Lv B., Zhang X., Chen L., Zhu K., Wang Z., Li B., Xiao W. (2020). Guizhi Fuling capsule exhibits antidysmenorrhea activity by inhibition of cyclooxygenase activity. Evid.-Based Complement. Alternat. Med..

